# Brucella infection combined with Nocardia infection: A case report and literature review

**DOI:** 10.1515/biol-2022-0815

**Published:** 2024-02-03

**Authors:** Yan Feng, Chun-Lei Zuo, Jia-Xin Shi

**Affiliations:** Department of Pulmonary and Critical Care Medicine, Lianyungang Clinical College of Nanjing Medical University, Lianyungang First People’s Hospital, 6 East Zhenhua Road, Lianyungang, 222006, China; Laboratory Department, Lianyungang Clinical College of Nanjing Medical University, Lianyungang First People’s Hospital, 6 East Zhenhua Road, Lianyungang, 222006, China

**Keywords:** brucellosis, nocardiosis, atypical pathogen, co-infection, pulmonary infection

## Abstract

Human brucellosis is an infectious disease caused by Brucella and is often misdiagnosed for atypical manifestations including fever of unknown origin, headache, weakness, among else. Nocardiosis is a zoonotic disease caused by the genus Nocardia, which usually spreads through the respiratory tract, skin, and digestive tract. Limited research has documented cases of co-infection involving both Brucella and Nocardia pathogens in patients. A 55-year-old male was admitted to our hospital with intermittent high-grade fever. Following sputum and blood cultures, as well as other laboratory examinations, the patient was diagnosed with concurrent brucellosis and nocardiosis. According to recommendations of previous studies and reports, the patient was successively treated with levofloxacin, doxycycline, piperacillin sodium and sulbactam sodium, trimethoprim-sulfamethoxazole, rifampicin, and tigecycline, after which the patient recovered and was discharged. Brucella and Nocardia are both opportunistic pathogens and simultaneous infection of Brucella and Nocardia is relatively rare. If patients continue to experience persistent fever despite receiving empirical antibiotic therapy, it becomes necessary to conduct examinations to identify potential atypical pathogens, including Brucella and Nocardia. Sputum staining, sputum culture, and blood culture are critical auxiliary examinations during clinical practice. The treatment plan should be selected based on guidelines and the individual patient’s condition. Regular reevaluation should be conducted, and antimicrobial agents should be adjusted accordingly.

## 1 Background

Brucella, a type of gram-negative bacterium, usually causes epidemics in cattle, sheep, and pigs. Humans may be infected when they come in contact with infected animals or ingest their meat or unpasteurized milk. Brucellosis is one of the most common infectious diseases causing fever of unknown origin [1], with a high misdiagnosis rate.

Nocardia lives in the soil and is gram-positive branching rod bacterium, entering the human body through respiratory tract, skin, or digestive tract [2]. The occurrence and transmission of nocardiosis are closely related to the body’s immunity.

Co-infection with both Brucella and Nocardia is not commonly encountered in clinical practice. The aim of this article is to provide some diagnostic and therapeutic strategies for clinicians managing such cases.

## 2 Case presentation

A 55-year-old male was admitted to our hospital with intermittent fever for the past month. The patient had fever a month ago, with the highest temperature of 40.4°C, accompanied by chills and weakness. He had a small amount of thick sputum, without sore throat, myalgia, or other symptoms. He provided a medical history of previous cerebral infarction and right eye surgery, without lingering residual sequelae. Of note, the patient cleaned a cowshed 4 months ago and was exposed to rain prior to the onset of the illness. He did not have a history of chronic obstructive pulmonary disease or any other chronic lung conditions. He denied any history of using immunosuppressive drugs or any other long-term medications.

**Figure 1:**
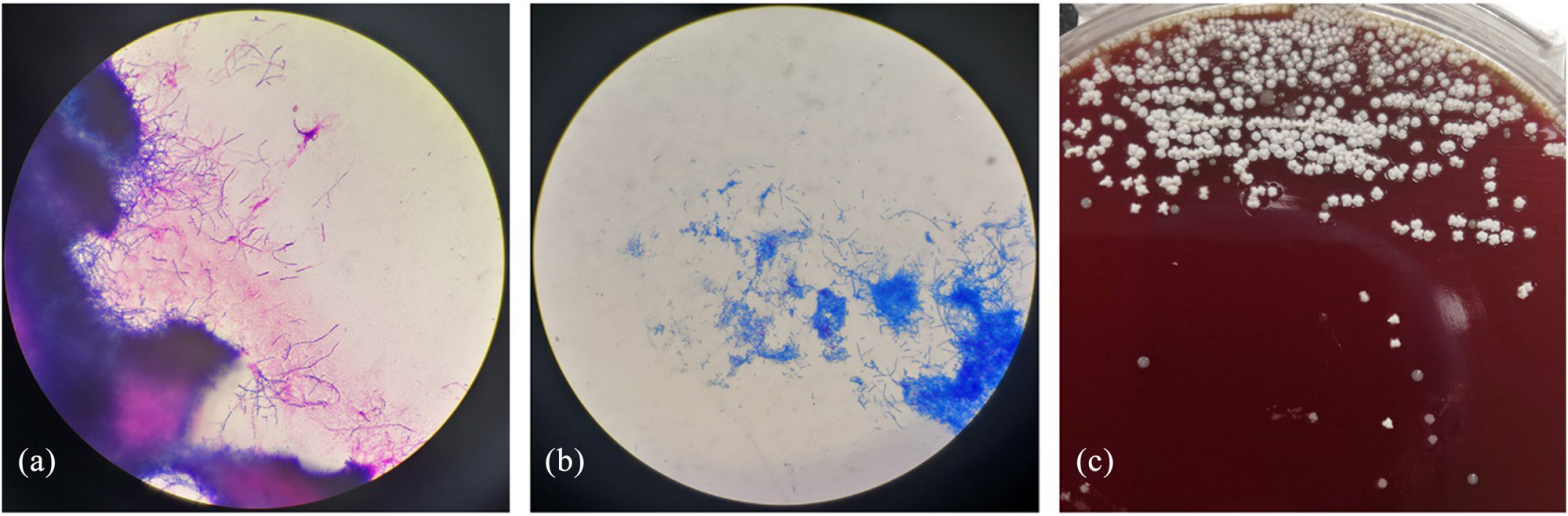
Sputum sample test. (a) Gram staining of the sputum sample. (b) Modified acid-fast staining of the sputum sample. (c) White dry colonies on blood agar plate.

**Figure 2:**
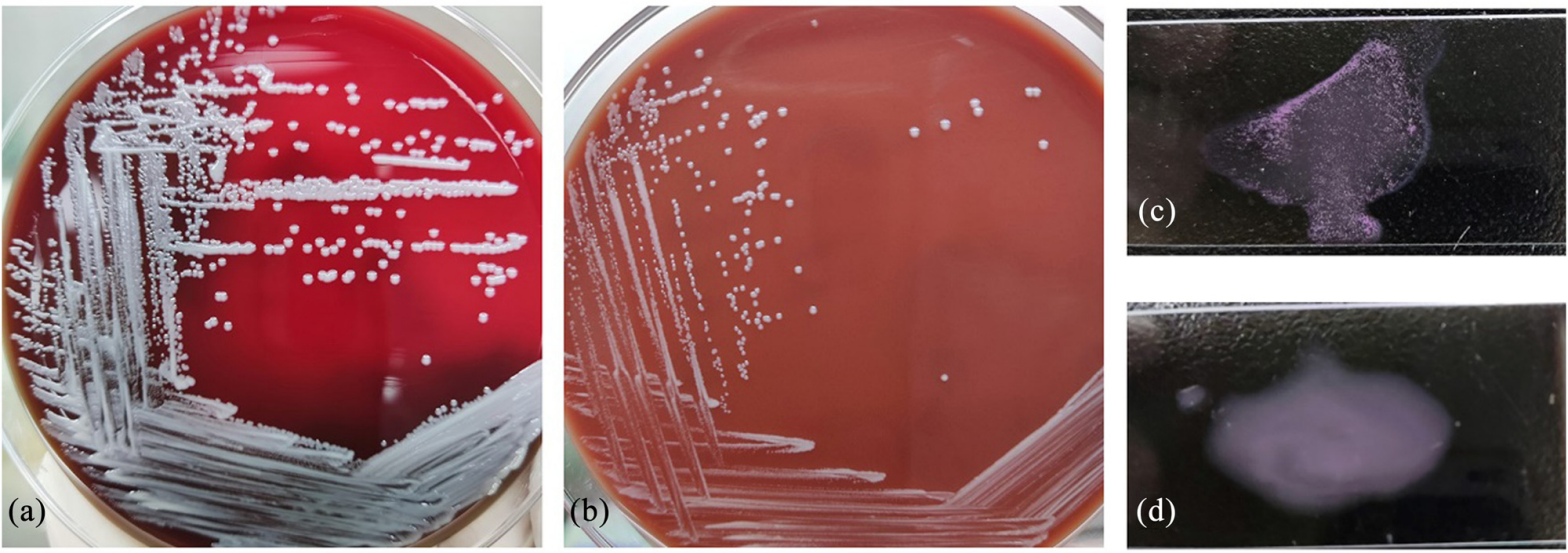
Blood sample test. (a) White dry colonies on blood agar plate. (b) White dry colonies on chocolate agar plate. (c) The patient’s serum sample: RBT showing positive. (d) The control’s serum sample: RBT showing negative.

**Figure 3:**
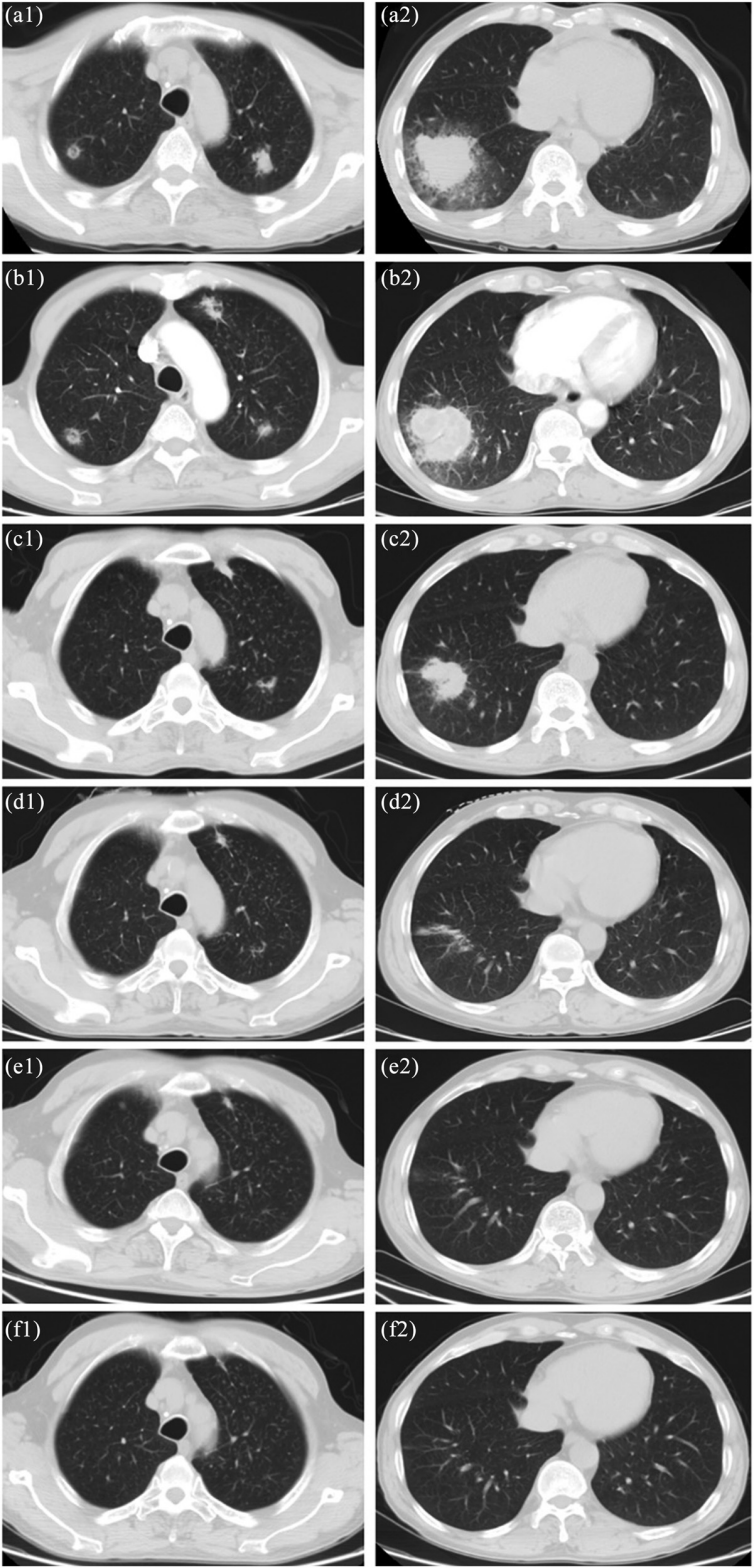
Chest CT scan. (a) CT scan on March 15th (day 1) showed occupying lesions in the RLL, multiple nodules in both upper lobes. (b) CT scan on March 23rd (day 9). (c) CT scan on April 1st (day 18) showed that lesions occupying both the RLL and multiple nodules in upper lobes shrank obviously. (d) CT scan on May 12th (day 59). (e) CT scan on Jun 18th (day 96). (f) CT scan on August 27th (day 166) showed almost complete absorption of lesions in both lungs.

On admission, his body temperature was 37.8°C he received ibuprofen suspension before hospitalization), heart rate was 110 bpm, respiration rate was 20 breaths/min, and blood pressure was 96/67 mmHg. We had not observed any signs of arthritis, lymphadenopathy, or hepatosplenomegaly in him. The patient demonstrated normal muscle strength and tone in all extremities, with negative pathological reflexes and no signs of meningeal irritation. There were no other remarkable findings on physical examination. After admission, the patient received routine examinations. His complete blood count showed a white blood cell count of 3.52 × 10^9^/L, red blood cell count of 2.85 × 10^12^/L, lymphocyte count of 1.1 × 10^9^/L, and a platelet count of 89 × 10^9^/L. Other laboratory examinations showed high erythrocyte sedimentation rate of 69 mm/h, increased level of C-reactive protein of 159.82 mg/L (0.00–5.00 mg/L). Serum biochemical index test revealed 2.65 mmol/L potassium, 26.7 g/L albumin, slightly elevated alanine aminotransferase (84 U/L), and aspartate aminotransferase (84 U/L), with normal creatinine. The examinations for autoimmune antibodies were negative. Immune globulin levels were normal. The human immunodeficiency virus antibody test was negative. Serum galactomannan and (1,3)-β-d-glucan tests were negative. T-SPOT.TB assay was negative. Tuberculosis and fungal infections were not found in the sputum smear. Sputum smear showed gram-positive filamentous bacilli resembling Nocardia or Actinomycetes (Figure 1a and b). All sputum specimens submitted for examination were deep purulent sputum expectorated by patient after thorough mouth rinsing. The sputum culture on day 4 of incubation grew Nocardia otitidiscaviarum (Figure 1c). A positive culture result for Brucella was obtained by blood culture on day 5 (Figure 2a and b), which was later confirmed by Rose Bengal plate agglutination test (RBT) (Figure 2c) and mass spectrometric detection and turned out to be Brucella melitensis. Echocardiogram was performed and revealed no evident anomalies. Chest computed tomography (CT) was performed and showed occupying lesions in the right lower lobe (RLL), multiple nodules in both upper lobes, miliary nodules in both lungs, and bilateral pleural effusion (Figure 3a).

During the first 2 days, we empirically gave the patient broad-spectrum antibiotic levofloxacin and symptomatic treatment, after which the patient still had fever. Combined with the patient’s persistent fever, imaging features, and the history of cleaning the cattle pen, on day 3, we adjusted the anti-infection treatment as follows: doxycycline hydrochloride enteric capsule plus piperacillin sodium and sulbactam sodium. When Nocardia infection was confirmed on day 4, oral trimethoprim-sulfamethoxazole (TMP-SMZ) was applied. The piperacillin sodium and sulbactam sodium were discontinued and rifampicin was applied after Brucella infection was confirmed on day 5. After the above treatments, the patient’s body temperature decreased slowly during the initial 4 days but still failed to return to normal and stayed at about 37.6°C in the next 3 days. In addition, the follow-up chest CT on day 9 (Figure 3b) showed no significant differences compared to previous results. Therefore, based on previous studies, we replaced doxycycline with tigecycline, which demonstrated superior antimicrobial efficacy [3,4]. The patient’s body temperature thereafter gradually decreased to normal and cough relieved obviously. On day 18, the chest CT (Figure 3c) showed that lesions occupying both the RLL and multiple nodules in upper lobes shrank obviously, and blood cultures on day 21 and day 25 were both negative. Therefore, the therapeutic regimen was adjusted to doxycycline plus TMP-SMZ. After 24 days of treatment, the sputum and blood cultures of the patient turned negative, and he was subsequently discharged from our hospital. After discharge, the patient continued to take doxycycline and rifampicin for 1 month and TMP-SMZ for 5 months. At the fifth month follow-up, the nodules on chest CT disappeared entirely (Figure 3f).


**Informed consent:** Informed consent has been obtained from all individuals included in this study.
**Ethical approval:** The research related to human use has been complied with all the relevant national regulations, institutional policies and in accordance with the tenets of the Helsinki Declaration, and has been approved by the authors' institutional review board or equivalent committee.

## 3 Discussion

Brucellosis is widely distributed in Asian countries including China, wherein the northern regions of China were reported facing more epidemics than the southern regions of China [5]. The manifestations of brucellosis mainly include fever, mostly characterized by wavelike fever, sweats, myalgia, fatigue, arthritis, and myocarditis [6,7]. So far, there has been no acknowledged standard of diagnosis for brucellosis. Combined with the epidemiological history, bacterial culture, and serological test results, a relatively high diagnostic rate can be achieved [8]. Patients usually have a history of contact with livestock or livestock products with Brucella infection. Common serological tests include RBT, standard agglutination test, enzyme-linked immunosorbent assay, 2-mercaptoethanol test, of which RBT is the most commonly used in clinical practice due to its high sensitivity, affordability, and rapid detection speed [9]. After further history inquiry, the patient in this case admitted sweeping the cattle pen prior to fever. We conducted sputum culture and blood culture, respectively, and ultimately found Brucella in the blood, following which RBT was carried out and presented a positive result. However, we failed to isolate Brucella from sputum throughout the course of disease. In addition, the patient refused the fiberoptic bronchoscopy as we suggested for bronchoalveolar lavage fluid detection. Combination treatment regimens were reported to prevent brucellosis relapse and avert complications more effectively than monotherapy [10]. There are lots of treatment regimens reported in the study by Skalsky et al. [11], and the recommended treatment duration is 1–3 months [12]. In this case, we chose doxycycline plus rifampicin for treatment initially. The patient’s fever symptom still existed and chest CT showed no obvious change after 7 days of doxycycline treatment. There are literature reports that tigecycline might demonstrate superior therapeutic efficacy in the treatment of brucellosis [3,4]. Therefore, based on the patient’s manifestations, we replaced doxycycline with tigecycline. The patient’s temperature returned to normal, blood culture turned negative as well as lung lesions shrank gradually (Figure 3), thus indicating that the treatment was successful. Therefore, we can conclude that tigecycline could be administered during the initial stage of high pathogen load, and when the condition stabilizes at a later stage, it can be switched to a conventional doxycycline sequential therapy. Regrettably, we did not conduct a follow-up blood culture examination before switching to tigecycline.

Nocardia, an opportunistic bacterium, usually invades immunocompromised hosts [13]. Despite the absence of common risk factors for Nocardia infection, such as HIV infection, chronic lung disease, and immunosuppressive medication use, the patient in this case was vulnerable to infection due to his advanced age and history of rain exposure prior to the onset of illness. Pulmonary nocardiosis is the most common presentation of Nocardia infection clinically, with nonspecific symptoms such as cough, dyspnea fever, and chest pain [14]. Chest imaging findings mainly include single or multiple nodules, pleural effusion, pulmonary infiltrates, and cavitary lesions [15], consistent with the findings on the patient’s chest CT. In addition, the central nervous system (CNS) is another common site of infection, often originating from pulmonary infection and presenting with insidious onset and high mortality rates [16]. Therefore, CNS Nocardiosis should be ruled out in every case of pulmonary nocardiosis especially with delayed response. The patient in this case underwent a cranial magnetic resonance imaging scan at a local hospital about a week before admission, which indicated lacunar cerebral infarction and no other abnormalities were found. The routine laboratory tests include gram staining, modified acid-fast staining, and germiculture [17]. Sputum smear of this patient showed gram-positive filamentous rods (Figure 1a), and modified acid-fast staining showed positive (Figure 1b). The following incubation confirmed the Nocardia otitidiscaviarum. Once antibiotics treatment is initiated, the diagnosis may become more difficult. In this situation, molecular biology methods such as Polymerase Chain Reaction and metagenomics next-generation sequencing should be taken into consideration [18]. Furthermore, we can consider lung biopsy if both of the above examination methods failed to find pathogenic bacteria. The most common treatment in clinical practice for Nocardia treatment is oral TMP-SMZ for 6 months [17]. However, there is variability in the sensitivity of Nocardia otitidiscaviarum to TMP/SMZ [19]. Nevertheless, due to the lack of drug sensitivity testing, we still decided to empirically use TMP/SMZ in this case, and, in the end, we obtained remarkable therapeutic results. Furthermore, tigecycline demonstrates efficacy against Brucella species and possesses certain antibacterial properties towards Nocardia [19].

## 4 Conclusion

Brucella and Nocardia are both opportunistic pathogens and simultaneous infection of Brucella and Nocardia is relatively rare. If patients continue to experience persistent fever despite receiving empirical antibiotic therapy, it becomes necessary to conduct examinations to identify potential atypical pathogens, including Brucella and Nocardia. Sputum staining, sputum culture, and blood culture are critical auxiliary examinations during clinical practice. The treatment plan should be selected based on guidelines and the individual patient’s condition. Regular reevaluation should be conducted, and antimicrobial agents should be adjusted accordingly.
